# Long-term exposure to transportation noise and diabetes mellitus mortality: a national cohort study and updated meta-analysis

**DOI:** 10.1186/s12940-024-01084-0

**Published:** 2024-05-04

**Authors:** Danielle Vienneau, Benedikt Wicki, Benjamin Flückiger, Beat Schäffer, Jean Marc Wunderli, Martin Röösli

**Affiliations:** 1 Department of Epidemiology and Public Health, Swiss Tropical and Public Health Institute, Kreuzstrasse 2, Allschwil, CH-4123 Switzerland; 2 University of Basel, Basel, Switzerland; 3 Swiss Federal Laboratories for Materials Science and Technology, Laboratory for Acoustics/Noise Control, Empa, Dübendorf, Switzerland

**Keywords:** Road traffic noise, Railway noise, Aircraft noise, Metabolic disease, Incidence, Review, meta-analysis

## Abstract

**Background:**

Long-term exposure to transportation noise is related to cardio-metabolic diseases, with more recent evidence also showing associations with diabetes mellitus (DM) incidence. This study aimed to evaluate the association between transportation noise and DM mortality within the Swiss National Cohort.

**Methods:**

During 15 years of follow-up (2001–2015; 4.14 million adults), over 72,000 DM deaths were accrued. Source-specific noise was calculated at residential locations, considering moving history. Multi-exposure, time-varying Cox regression was used to derive hazard ratios (HR, and 95%-confidence intervals). Models included road traffic, railway and aircraft noise, air pollution, and individual and area-level covariates including socio-economic position. Analyses included exposure-response modelling, effect modification, and a subset analysis around airports. The main findings were integrated into meta-analyses with published studies on mortality and incidence (separately and combined).

**Results:**

HRs were 1.06 (1.05, 1.07), 1.02 (1.01, 1.03) and 1.01 (0.99, 1.02) per 10 dB day evening-night level (L_den_) road traffic, railway and aircraft noise, respectively (adjusted model, including NO_2_). Splines suggested a threshold for road traffic noise (~ 46 dB L_den_, well below the 53 dB L_den_ WHO guideline level), but not railway noise. Substituting for PM_2.5_, or including deaths with type 1 DM hardly changed the associations. HRs were higher for males compared to females, and in younger compared to older adults. Focusing only on type 1 DM showed an independent association with road traffic noise. Meta-analysis was only possible for road traffic noise in relation to mortality (1.08 [0.99, 1.18] per 10 dB, *n* = 4), with the point estimate broadly similar to that for incidence (1.07 [1.05, 1.09] per 10 dB, *n* = 10). Combining incidence and mortality studies indicated positive associations for each source, strongest for road traffic noise (1.07 [1.05, 1.08], 1.02 [1.01, 1.03], and 1.02 [1.00, 1.03] per 10 dB road traffic [*n* = 14], railway [*n* = 5] and aircraft noise [*n* = 5], respectively).

**Conclusions:**

This study provides new evidence that transportation noise is associated with diabetes mortality. With the growing evidence and large disease burden, DM should be viewed as an important outcome in the noise and health discussion.

**Supplementary Information:**

The online version contains supplementary material available at 10.1186/s12940-024-01084-0.

## Introduction

Ranked, world-wide, as the fourth most common non-communicable disease in terms of premature mortality, diabetes mellitus (DM) has a large disease burden [1]. While the main modifiable risk factors for cardio-metabolic diseases relate to lifestyle including diet, alcohol, smoking and exercise [2], understanding the role of environmental stressors including noise is important, especially since so many persons are chronically exposed to noise levels known to be harmful [3]. The global increase in DM prevalence [4] in tandem with a shift toward increased urbanization drive this need.

It is through two main mechanisms that transportation noise is posited to be harmful to the metabolic system. First is via a physiological stress response in which noise exposure activates the sympatico-adrenal and the hypothalamic-pituitary-adrenal (HPA) axes [5, 6]. The physiological cascade leads to a release of stress hormones and dysregulating hormones that control appetite. The related stress responses lead to inflammation and oxidative stress [7, 8] which are known to contribute to disturbed glucose metabolism and increased risk for type 2 DM [9]. The second mechanism is via sleep disturbance caused by noise [10, 11] that contributes to impaired glucose regulation, cortisol release and dysregulation of leptin, altering metabolism and increasing the risk for central obesity [12–15]. Further, as a general stressor [16, 17], transportation noise can negatively influence lifestyle choices and exacerbate modifiable risk factors linked to cardio-metabolic disease, such as physical inactivity [18, 19], smoking and alcohol consumption [20], obesity [21], and hypertension [22, 23].

DM was identified as an important health outcome in the 2018 WHO Environmental Noise Guideline (ENG) systematic reviews [23, 24]; though at the time the sparse body of evidence comprised of only one cohort study from Denmark [25]. It reported an association for incident diabetes of 1.08 (95% confidence interval (CI): 1.02, 1.14) per 10 dB L_den_, between 50 and 70 dB road traffic noise [25], and the WHO review concluded the need for more research. Since then evidence supporting an association between transportation noise and type 2 diabetes mellitus (DM) incidence has increased with studies from Europe and North America [26–30]. Initial meta-analyses following the original WHO review have since been conducted. For example, Zare Sakhvidi et al. [31] reported the risk increase for DM in relation to all transportation noise as 1.06 (1.03, 1.09) per 5 dB L_den_ based on 9 studies, including longitudinal and cross-sectional designs. Stratified by source, the associations were strongest for aircraft noise (1.17 [1.06, 1.29]), followed by road traffic noise (1.07 [1.02, 1.12]). Building on that, and including 6 studies until March 2019 focused on incidence, Vienneau et al. [32] reported a RR of 1.08 (1.02, 1.15) per 10 dB L_den_ for all transportation noise combined, 1.20 (0.88, 1.63) for aircraft noise, and 1.11 (1.08, 1.15) for road traffic noise. Both of these meta-analyses reported no association for railway noise, though based only on 2 studies these findings were inconclusive. Also, neither included studies on mortality due to a lack of available studies at the time. Given that DM can lead to serious health complications, impacting quality of life and contributing to premature mortality [4, 33], we undertook this study to fill this gap.

This study aimed to evaluate the association between road traffic, railway and aircraft noise exposure and DM mortality in a nation-wide cohort in Switzerland with high quality exposure assessment and 15 years of follow-up. A secondary aim was to incorporate these results and the latest findings from the literature into an updated meta-analysis.

## Methods

### Study population

The study includes nearly all adults living in Switzerland, following them from 01 January 2001 to 31 December 2015. It used the Swiss National Cohort (SNC), that links the national census with the births, mortality (providing date and cause of death) and emigration registries [34, 35]. Over 98% of the population are captured due to mandatory participation in the 04 December 2000 census [36]. Approval for the SNC was granted by the Ethics Committees of the Cantons of Zurich and Bern. After removing those under 30 years of age at baseline (to obtain the adult population over 30 years) and those with incorrect historical linkages (33.4 and 8.2% total population, respectively), the study sample included 4.4 million eligible individuals. Ultimately 4.1 million were retained after excluding those without a residential coordinate or living in institutions (4.8%), individual-level education and/or socio-economic position (2.2%), or exposure data (0.2%) (Supp. Table S1).

### Outcome definition

Outcomes were defined considering DM, indicated on the death certificate, as the primary definitive cause of death, concomitant, consecutive or initial disease. The main analyses used non-type 1 diabetes mellitus (i.e. nonT1-DM) as the outcome, defined via ICD10 codes E11-E14. Type 1 diabetes (E10) is distinct from other types of DM as it is an autoimmune disease less dependent on behavioral factors and is typically diagnosed before adulthood. Two secondary analyses were thus conducted to consider Type 1 DM by: (i) expanding the case definition to E10-14 (i.e. DM), and (ii) investigating Type 1 DM using only E10 (i.e. T1-DM).

### Exposure assessment

Separate estimates for road traffic, railway and aircraft noise levels, modelled at the façade, were available from the SiRENE project (Short and Long Term Effects of Transportation Noise Exposure) for the year 2001 and 2011 [37, 38]. For road traffic and railway noise, the source models sonROAD [39] and sonRAIL [40] were combined with the sound propagation models of the corresponding predecessor models, StL-86 [41] and SEMIBEL [42]. Aircraft noise of the three Swiss civil airports (Zürich, Geneva and Basel) and the Payerne military airfield was calculated using FLULA2 [43]. Noise levels for each source were quantified using the L_den_ (i.e. weighted logarithmic mean of L_eq, day_, L_eq, evening_ and L_eq, night_ with a penalty of 5 dB for evening hours and 10 dB for night hours). Intermittency ratio (IR, expressed as a percent), that describes the eventfulness of the noise situation [44], was also available. When no single events can be perceived above background noise the IR is 0%, while 100% indicates that all of the noise energy is from distinct events. Following our previous studies, IR at night (23 to 7 h) from all transportation sources combined was used [45, 46]. The database also included counts of nighttime noise events (vehicle pass-bys that exceed background noise levels by at least 3 dB L_eq_), here summed for all transportation sources combined and referred to as number of events.

Given the up to 15 years of follow up, and availability of both noise exposure and residential geocode at 2001 and 2011, exposure was assigned at the start of each of the following 5-year periods: 2001–2005, 2006–2010 and 2011–2015. Direct temporal matches were available for the first and last period, and the following workflow was used for the middle period (2006–2010). First, movers were identified using moving date (if available) and the 2010 census question “living in the same community 5 years before”. Next, non-movers and anyone moving after 2006 were assigned the 2001 noise estimates, while anyone moving prior to 2006 were assigned the 2011 noise estimates (16.5%).

Using the geocoded home location and floor of residence for individuals in the SNC, noise exposure (L_den_, dB) at the maximum exposed façade was extracted from the SiRENE database (along with the nighttime IR and number of events for the same façade point). Noise estimates from the middle floor were taken if explicit information on floor of residence was missing in the SNC [45, 47]. To avoid unreasonably low exposures to a specific transportation source that would not be distinguishable from background noise from diffuse sources, L_den_ values below 35 dB (road traffic noise) or 30 dB (railway and aircraft noise) were set to these threshold levels, i.e., set to 35 and 30 dB, respectively [48]. A subset analysis for aircraft noise involved restricting the population to those with aircraft noise exposure L_den_ > 30 dB.

Annual mean air pollution concentrations for year 2010 were extracted at the home location from 100 × 100 m hybrid land use regression models for Europe available from the ELAPSE project [49]. Both NO_2_ and PM_2.5_ (µg/m^3^) were extracted and used separately, with NO_2_ considered in the main model as the better marker for traffic-related air pollution [50]. Air pollution was included as an adjustment due to the potential for confounding, i.e. because both noise and air pollution derive from transportation [51, 52].

### Statistical analysis

The Cox proportional hazards model was used to evaluate associations between DM mortality and each noise source (road traffic, railway and aircraft included in the same model). The timescale was age, and the model was stratified by sex. A time-varying approach was used to incorporate residential history during follow-up, and to account for time trends in noise exposure and mortality. Treated as a closed cohort (starting on 01 January 2001), individuals were followed until the date of the event, emigration, death from another cause or end of follow-up on 31 December 2015. Hazard ratios (HR) and 95% confidence intervals were reported per 10 dB L_den_. Additionally, splines with 3 degrees of freedom were used to model the association and allow a potential non-linear relationship.

The following individual-level covariates were available in the SNC and used as adjustments in the models. Sex (female/male), civil status (single, married, widowed, divorced; available 2001 and 2011), education level (compulsory or less, upper secondary level, tertiary level), mother tongue (German and Rhaeto-Romansch, French, Italian, other), nationality (Swiss, non-Swiss; available 2001 and 2011) and local index of socio-economic position (SEP in quartiles; calculated in a moving window approach by Panczak et al. [53] as the average of the 50 nearest neighbours; available 2001 and 2011). Additionally, area-level SEP and unemployment rate were calculated at the community (*n* = 2896 in 2001, *n* = 2585 in 2011) and regional level (*n* = 26, i.e. Swiss cantons) (available 2001 and 2011). Where available, these were updated at the start of period 3 using the 2011 values otherwise the baseline values were retained. As spatial covariates, the area-level SEP measures were also updated for movers at 2006.

An incremental adjustment strategy was used in specifying the models. The base model (Model 0) included road traffic, railway and aircraft noise, age as time scale, and strata sex and 5-year period. Next the individual-level covariates (civil status, education level, mother tongue, nationality and local-SEP) were added in Model 1, followed by all area-level covariates (area-SEP and unemployment rate) in Model 2. Air pollution was added next, with Model 3 including NO_2_ and designated the main model, and Model 3b as the alternative with PM_2.5_. As a last step, quartiles of noise eventfulness at night for total transportation noise was added, as IR in Model 4.1 or number of events in Model 4.2. The variance inflation factor (VIF < 5) was calculated *post hoc* to evaluate the potential multicollinearity between exposure variables [54, 55]. In addition, age (three groups: 30–65, 65–80 and over 80 years) and sex specific models were calculated for the main Model 3. Analyses were conducted in Stata16 (Stata Corp LLC) with plots developed in R (version4.0; R Development Core Team).

### Meta-analysis

 The results from this study were added to a meta-analysis developed and regularly updated to support health impact assessment efforts in Switzerland [32]. An adapted OVID search on diabetes from van Kempen et al. [23] for the WHO ENG review was used (see Supp. Meta-analysis Sect. 1.1 for search string). Here the search was repeated to include studies until 22 November 2023. The search was supplemented with recent publications maintained in the author’s collections, identified previously through hand searching and/or email alerts. Studies on road traffic, railway or aircraft noise exposure and incidence and mortality of diabetes (excluding gestational diabetes) were retained, while those only reporting prevalence were excluded (see Supp. Meta-analysis Sect. 1.2 for inclusion/exclusion criteria). Risk estimates adjusted for air pollution were selected if available. If there was more than one air pollution adjusted model, the NOx-adjusted was selected over the PM-adjusted on the basis of NO_2_ being the better proxy for traffic related air pollution. Studies from different cohorts within the same country were allowed, so long as the exact cohort was not double counted. Pooled exposure-response associations were calculated by source (road traffic, railway, aircraft noise, and an overall combined), and by disease state (incidence, mortality and an overall combined), using random-effects meta-analysis and presented graphically in Forest plots. Sensitivity analyses were used to assess the influence of overlaps from multiple cohorts in the same country by retaining the largest study only within strata (noise source or disease state, as appropriate). Analyses were conducted in Stata16 (Stata Corp LLC).

## Results

### Study population

Based on the resident population in Switzerland on 01 January 2001, the cohort included 4,136,220 adults aged 30 years who met the inclusion criteria. In total 56 million person-years were accrued over 15 years of follow up (mean 13.4 years) ending on 31 December 2015. The number of recorded deaths was 72,342 (1.7%) for nonT1-DM, 1046 (0.03% for T1-DM, 73,388 (1.8%) for total DM deaths. As a nation-wide administrative cohort, the population at baseline largely comprised Swiss nationals and those with German as their mother tongue (83.2 and 65.5%, respectively). A large proportion were also well educated and married (75.3 and 69.9%, respectively). Compared to the whole cohort, a higher proportion of DM deaths were in the retired age group of 65–79 years, lowest educated, widowed, and in men. The distributions of socioeconomic status and exposures were also comparable between the cohort and DM deaths (Table 1).

**Table 1:** Full population characteristics at baseline, 2001

Characteristic	Cohort	Deaths^c^		
		nonT1-DM	DM	T1-DM
Number of participants	4,136,220	72,342	73,388	1,046
Person-years	55,563,446			
Male (%)	47.8	52.3	52.4	58.4
Age (%)				
30–64	76.5	22.1	22.5	53.0
65–79	18.3	55.3	55.0	34.3
80+	5.2	22.6	22.5	12.7
Mother tongue (%)				
German and Rhaeto-Romansch	65.5	72.5	72.4	70.4
French	19.3	17.2	17.2	20.4
Italian	7.4	7.9	7.9	5.7
Other	7.8	2.4	2.4	3.5
Education (%)				
Compulsory education or less	24.7	44.2	44.0	30.2
Upper secondary level	53.0	44.4	44.5	50.6
Tertiary level education	22.3	11.4	11.5	19.2
Marital status (%)^a^				
Single	13.4	8.4	8.6	16.2
Married	69.9	57.0	57.0	57.4
Divorced	8.5	7.6	7.7	12.1
Widowed	8.2	26.9	26.7	14.3
Swiss nationality (%)^a^	83.2	91.5	91.5	89.9
Local-SEP (%), mean (SD)^a, b^	63.3 (10.5)	61.8 (10.0)	61.8 (10.0)	63.3 (10.2)
Area-SEP community (%), mean (SD)^a^	62.9 (6.7)	62.4 (6.5)	62.4 (6.5)	63.2 (6.4)
Area-SEP community-region (%), mean (SD)^a^	0.1 (5.2)	-0.4 (5.1)	-0.4 (5.1)	0.1 (5.0)
Area unemployment community (%), mean (SD)^a^	3.5 (1.4)	3.5 (1.4)	3.5 (1.4)	3.6 (1.4)
Area unemployment community-region(%), mean (SD)^a^	0.0 (1.2)	0.1 (1.2)	0.1 (1.2)	0.1 (1.2)
Road traffic noise, Lden (dB), mean (SD)^a^	54.3 (8.2)	55.0 (8.2)	55.1 (8.2)	55.4 (8.0)
Railway noise Lden (dB), mean (SD)^a^	38.4(11.0)	38.9 (11.3)	38.9 (11.3)	39.0 (11.3)
Aircraft noise Lden (dB), mean (SD)^a^	34.5 (7.7)	34.2 (7.5)	34.2 (7.5)	34.5 (7.4)
Total noise Intermittency Ratio (IR) at night (%)^a^	69.7 (20.9)	70.3 (20.4)	70.3 (20.4)	69.6 (19.9)
Total noise events at night (count), mean (SD)^a^	171.1 (188.7)	181.5 (196.6)	181.7 (196.8)	190.8 (205.9)
NO_2_ concentration (µg/m^3^), mean (SD)	23.6 (7.4)	23.7 (7.5)	23.8 (7.5)	24.6 (7.6)
PM_2.5_ concentration (µg/m^3^), mean (SD)	15.9 (2.4)	15.9 (2.4)	15.9 (2.4)	16.1 (2.3)

^a^census/exposure data available at multiple time points, and updated at beginning of each 5-year period; baseline values shown here

^b^Quartiles of local-socioeconomic position (SEP) used in models

^c^Outcome definition: nonT1-DM (ICD-10: E11-E14); DM (IDC-10: E10-E14, includes type 1 diabetes); T1-DM (ICD-10: E10, type 1 diabetes)

Noise models for the year 2001 and 2011 were used in the exposure assessment, with high correlations between the years at the same location i.e. for non-movers (Pearson’s *r* = 0.91 for aircraft noise, 0.95 for railway, and 0.97 for road traffic noise; data not shown). For each noise source and metric, the correlations in the full cohort were moderate to high across the three 5-year periods that exposure was assigned (Pearson’s *r* ≥ 0.83 for aircraft noise; *r* ≥ 0.67 for railway and road traffic noise; and *r* ≥ 0.56 for nighttime IR and number of events) (Supp. Figure S1). Correlations between noise metrics within a period were low (*r* < 0.2), except for between road traffic noise and the number of nighttime events (*r* ≤ 0.63). Correlations between the air pollutants and noise metrics were low to moderately low (*r* = 0.05–0.41), and mostly lower for PM_2.5_ than NO_2_. The proportion of noise exposures set to the *a-priori* minimum (or censored) level were 1.4, 46.1 and 67.6% for road traffic, railway and aircraft noise, respectively.

### Main findings

In the main Model 3 (Table 2), adjusted for other noise sources, socio-economic indicators and NO_2_, the HR showed increased association with nonT1-DM mortality for road traffic noise (1.06 [1.05, 1.07] per 10 dB L_den_) and to a lesser extent railway noise (1.02 [1.01, 1.03] per 10 dB L_den_). The increase for aircraft noise was very small and only borderline significant (1.01 [0.99, 1.02] per 10 dB L_den_). These associations were robust to adjustment for PM_2.5_ in place of NO_2_ (Model 3b vs. Model 3), and when further adjusting for IR at night (Model 4.1 vs. Model 3). The L_den_ road traffic noise estimates attenuated, however, when adjusting for the number of noise events at night (Model 4.2 vs. Model 3). The HRs for the metrics related to eventfulness at night were consistent across quartiles of IR, with a small increased risk (e.g. 1.019 [1.018, 1.021] in Q4 vs. reference; Model 4.1), and increasing across quartiles for number of events (e.g. 1.051 [1.050, 1.052] in Q4 vs. reference; Model 4.2) (Supp. Table S2).

**Table 2:** Association between transportation noise source and diabetes mortality. Full cohort including 4,136,220 adults over 30 years, followed from 2001 to 2015. Multipollutant models, adjusting for the other two noise sources plus NO_2_. HR and 95% confidence intervals (CI) per 10 dB increase in Lden noise source

Outcome definition	Main: nonT1-DM			Secondary 1: DM			Secondary 2: T1-DM		
	ICD-10: E11-E14			IDC-10: E10-E14			ICD-10: E10		
N deaths	72,342			73,388			1046		
**Noise source**	**Road traffic**	**Railway**	**Aircraft**	**Road traffic**	**Railway**	**Aircraft**	**Road traffic**	**Railway**	**Aircraft**
Model 0	1.08 (1.07, 1.09)	1.04 (1.03, 1.05)	0.97 (0.96, 0.98)	1.08 (1.07, 1.09)	1.04 (1.03, 1.05)	0.97 (0.96, 0.98)	1.17 (1.09, 1.27)	1.04 (0.99, 1.10)	1.00 (0.92, 1.08)
Model 1	1.06 (1.05, 1.07)	1.03 (1.02, 1.03)	1.02 (1.01, 1.03)	1.06 (1.05, 1.07)	1.03 (1.02, 1.03)	1.02 (1.01, 1.03)	1.14 (1.06, 1.23)	1.04 (0.98, 1.10)	1.00 (0.92, 1.09)
Model 2	1.06 (1.05, 1.07)	1.02 (1.02, 1.03)	1.01 (1.00, 1.02)	1.06 (1.05, 1.07)	1.02 (1.02, 1.03)	1.01 (1.00, 1.02)	1.13 (1.04, 1.22)	1.02 (0.96, 1.08)	0.94 (0.84, 1.04)
Model 3 (main model)	1.06 (1.05, 1.07)	1.02 (1.01, 1.03)	1.01 (0.99, 1.02)	1.06 (1.05, 1.07)	1.02 (1.01, 1.03)	1.01 (0.99, 1.02)	1.09 (1.01, 1.18)	1.02 (0.96, 1.08)	0.92 (0.83, 1.03)
Model 3b	1.06 (1.05, 1.07)	1.02 (1.02, 1.03)	1.01 (0.99, 1.02)	1.06 (1.05, 1.07)	1.02 (1.02, 1.03)	1.00 (0.99, 1.02)	1.12 (1.03, 1.21)	1.02 (0.96, 1.08)	0.92 (0.82, 1.02)
Model 4.1	1.06 (1.05, 1.07)	1.02 (1.01, 1.03)	1.01 (0.99, 1.02)	1.06 (1.05, 1.07)	1.02 (1.01, 1.03)	1.01 (0.99, 1.02)	1.09 (1.00, 1.18)	1.01 (0.95, 1.08)	0.92 (0.83, 1.03)
Model 4.2	1.04 (1.03, 1.05)	1.02 (1.02, 1.03)	1.01 (1.00, 1.02)	1.04 (1.03, 1.06)	1.02 (1.02, 1.03)	1.01 (0.99, 1.02)	1.08 (0.97, 1.19)	1.03 (0.97, 1.09)	0.93 (0.83, 1.03)

Notes:

Model 0 included noise exposure (road traffic, railway and aircraft noise; Lden), strata sex and period (i.e. 2001–2005, 2006–2010, or 2011–2015)

Model 1 included Model 0 + the individual-level covariates civil status, education level, mother tongue, nationality and quartiles of local-SEP

Model 2 included Model 1 + community and regional SEP score and unemployment

Model 3 (main model) included Model 2 + quartiles of NO_2_ exposure

Model 3b included Model 2 + quartiles PM_2.5_ exposure

Model 4.1 included Model 3 + quartiles of intermittency ratio (IR) at night

Model 4.2 included Model 3 + quartiles of number of noise events at night

**Fig. 1:**
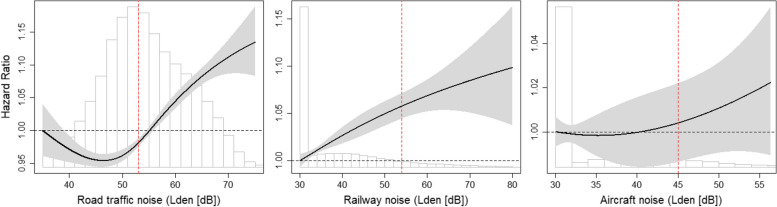
Natural splines (3 df) for the association between road traffic, railway or aircraft noise and nonT1-DM mortality. Full cohort including 4,136,220 adults over 30 years (and 72,342 nonT1-DM deaths), followed from 2001 to 2015. Multipollutant models, adjusting for the other two noise sources plus NO_2_ **Notes:** Outcome definition: nonT1-DM(ICD-10: E11-E14). Model 3: Natural spline (3 df) for road traffic, railway or aircraft noise (other two noise sources included as adjustment), included strata sex and period (i.e. 2001–2005, 2006–2010, or 2011–2015), and adjusted for mother tongue, nationality, civil status, education, local-SEP, area-SEP and unemployment, and NO_2_. Vertical red lines show WHO guideline levels based on Lden: road traffic = 53 dB, railway = 54 dB, aircraft = 45 dB

The exposure-response signaled a threshold for road traffic noise, with an approximatively linear risk increase starting from around 46 dB L_den_, while the risk for railway noise was approximatively linearly increasing from the lowest level (i.e., 30 dB L_den_). With wide confidence intervals, the exposure-response for aircraft noise indicated a null association, though with increasing trend (Fig. 1). Comparison of the AICs between the linear and non-linear models confirmed only the road traffic noise association deviated from linear (Supp. Table S3). For both road traffic and railway noise the associations were stronger in males compared to females, and stronger in younger compared to older adults (Table 3). Including type 1 diabetes (DM) did not change the results. Focusing only on those deaths in T1-DM patients showed a slightly stronger association for road traffic noise though with wide confidence intervals (1.09 [1.01, 1.18] per 10 dB L_den_) (Table 2). These associations for T1-DM patients were no longer significant after stratifying by age or sex (Table 3).

**Table 3:** Effect estimates by sex and specific age groups for the association between transportation noise source and diabetes mortality. Full cohort including 4,136,220 adults over 30 years, followed from 2001 to 2015. Multipollutant models, adjusting for the other two noise sources plus NO_2_. HR and 95% confidence intervals (CI) per 10 dB increase in Lden noise source

Outcome^a^	*N* deaths / Source	Sex^b^			Age^c^			
		Male	Female	*p*-interaction	30–64	65–79	80+	*p*-trend
	N deaths	37,842	34,500		15,982	39,997	16,363	
Main: nonT1-DM	Road traffic	1.07 (1.05, 1.08)	1.05 (1.03, 1.06)	< 0.0001	1.12 (1.09, 1.14)	1.05 (1.04, 1.07)	1.02 (1.00, 1.04)	< 0.0001
	Railway	1.03 (1.02, 1.04)	1.01 (1.00, 1.02)	< 0.0001	1.03 (1.01, 1.04)	1.03 (1.02, 1.04)	1.00 (0.99, 1.02)	< 0.0001
	Aircraft	1 (0.98, 1.01)	1.02 (1.00, 1.04)	0.0609	1.00 (0.97, 1.03)	1.01 (0.99, 1.03)	1.01 (0.99, 1.04)	0.4445
	N deaths	38,453	34,935		16,536	40,356	16,496	
Secondary 1: DM	Road traffic	1.07 (1.05, 1.08)	1.05 (1.03, 1.06)	< 0.0001	1.12 (1.09, 1.14)	1.05 (1.04, 1.07)	1.02 (1.00, 1.04)	< 0.0001
	Railway	1.03 (1.02, 1.04)	1.01 (1.00, 1.02)	< 0.0001	1.03 (1.01, 1.04)	1.03 (1.02, 1.04)	1.00 (0.99, 1.02)	< 0.0001
	Aircraft	1.00 (0.98, 1.01)	1.02 (1.00, 1.04)	0.0670	1.00 (0.97, 1.02)	1.01 (0.99, 1.03)	1.01 (0.98, 1.04)	0.4269
	N deaths	611	435		554	359	133	
Secondary 2: T1-DM	Road traffic	1.07 (0.96, 1.19)	1.12 (0.99, 1.27)	0.0300	1.09 (0.98, 1.22)	1.10 (0.95, 1.26)	1.08 (0.86, 1.36)	0.1000
	Railway	1.04 (0.97, 1.12)	0.98 (0.90, 1.08)	0.2700	1.03 (0.95, 1.11)	0.99 (0.89, 1.10)	1.01 (0.86, 1.18)	0.7400
	Aircraft	0.91 (0.79, 1.04)	0.95 (0.80, 1.12)	0.1228	0.84 (0.72, 0.98)	1.10 (0.93, 1.30)	0.81 (0.58, 1.12)	0.0156

Notes:

^a^Outcome definition: nonT1-DM (ICD-10: E11-E14); DM (IDC-10: E10-E14, includes type 1 diabetes); T1-DM (ICD-10: E10, type 1 diabetes)

^b^Sex groups included all adults over 30 years old

^c^Age groups included both sexes

Model 3 included noise exposure (road traffic, railway and aircraft noise; Lden), strata sex* and period (i.e. 2001–2005, 2006–2010, or 2011–2015), plus the individual-level covariates civil status, education level, mother tongue, nationality and quartiles of local-SEP, community and regional SEP score and unemployment, NO_2_ exposure. *only in models including all ages

### Subset analysis for aircraft noise

Approximately 30% of both the cohort and DM deaths were within the subset with aircraft noise exposure > 30 dB L_den_, and thus exposed to some aircraft noise. Aside from aircraft noise exposure, the characteristics of those in the subset were broadly similar to the full cohort (Table 1 vs. Supp. Table S4). Based on a linear model, the association within the subset remained null, similar to the full cohort (Supp. Table S3). In contrast, though still with rather wide confidence intervals, the spline suggested an increased risk between 40 and 50 dB before trailing off at higher levels (Supp. Figure S2).

### Meta-analysis

The search identified 21 eligible studies. After exclusions (i.e., resolving overlaps and replacements), 13 were retained, and considering the present study the total number was 14. Only two of the previously published studies were on mortality, both on road traffic noise [56, 57]. For details, see Supp. Meta-analysis Sect. 1.3–1.5 for the PRISMA diagram, characteristics of included studies, and list of excluded studies. All were cohorts, except the single case control study by Eriksson et al. [13]. Most were conducted in Europe [13, 27, 28, 30, 56–62], with two from Canada [26, 29]. Five studies including the present one, all from Denmark or Switzerland, considered other noise sources in the adjustment sets [27, 28, 30, 61]. All except two studies considered air pollution, while six could adjust for key lifestyle factors such as smoking, BMI, diet, alcohol consumption, and/or physical activity [27, 28, 58, 59, 61, 62]. Two countries had multiple cohorts and thus potential overlaps in the study populations. The studies from Switzerland included the SAPALDIA cohort investigating incidence [27] and this larger SNC study on mortality. Studies from Denmark included the Diet, Cancer and Health (DCH) cohort [28], the Danish (DK) Nurses Study including women only [59], the Danish National Health Survey (DNHS) [61] and the largest Danish National Cohort (DNC) [30], all investigating incidence, and the Danish Nurses Study also reporting on mortality [57].

**Fig. 2:**
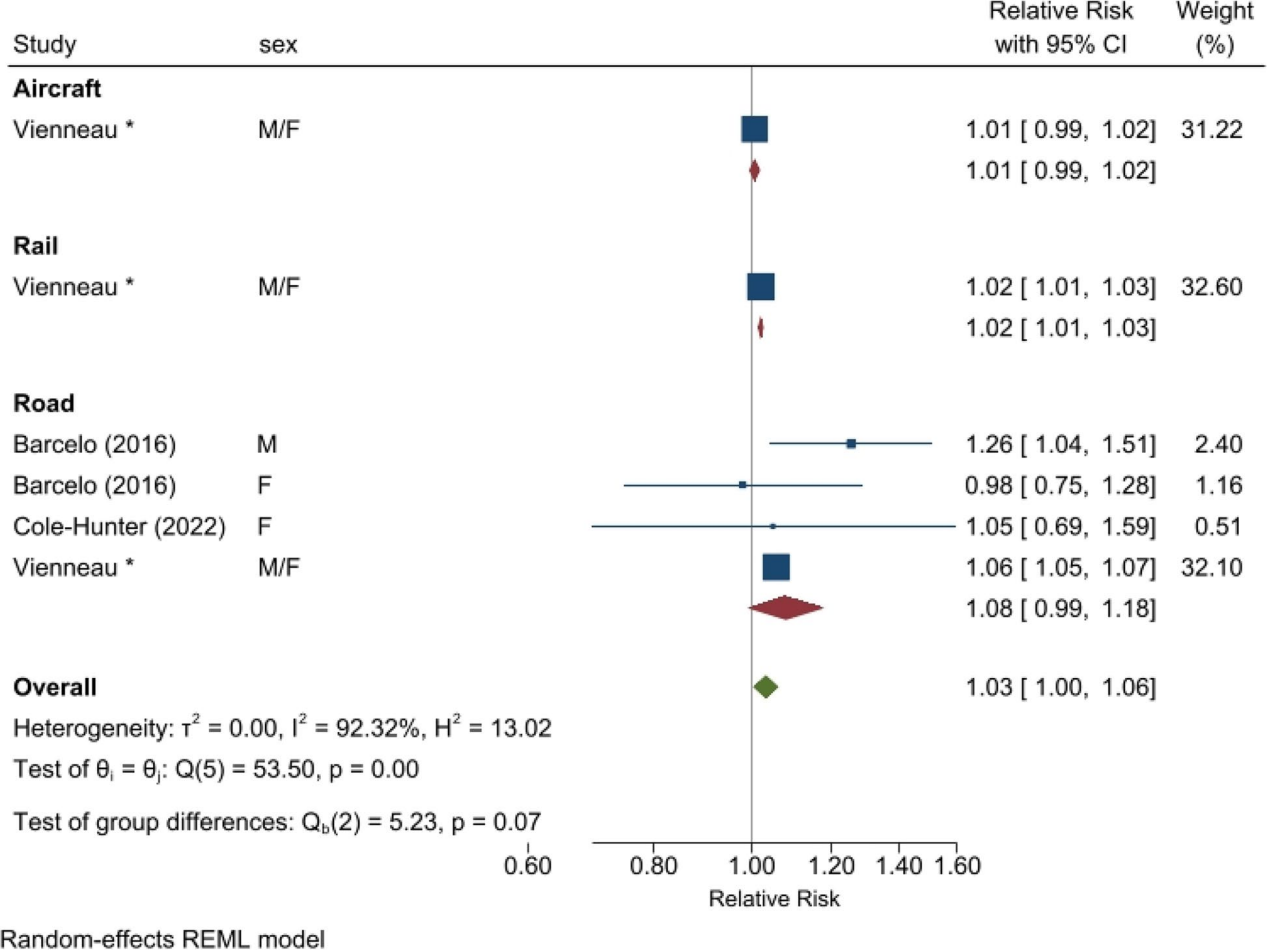
Meta-analysis for association between source-specific transportation noise and diabetes mortality **Notes:** “Vienneau*” refers to the results of this study (Table 2, Model 3 for nonT1-DM)

**Fig. 3:**
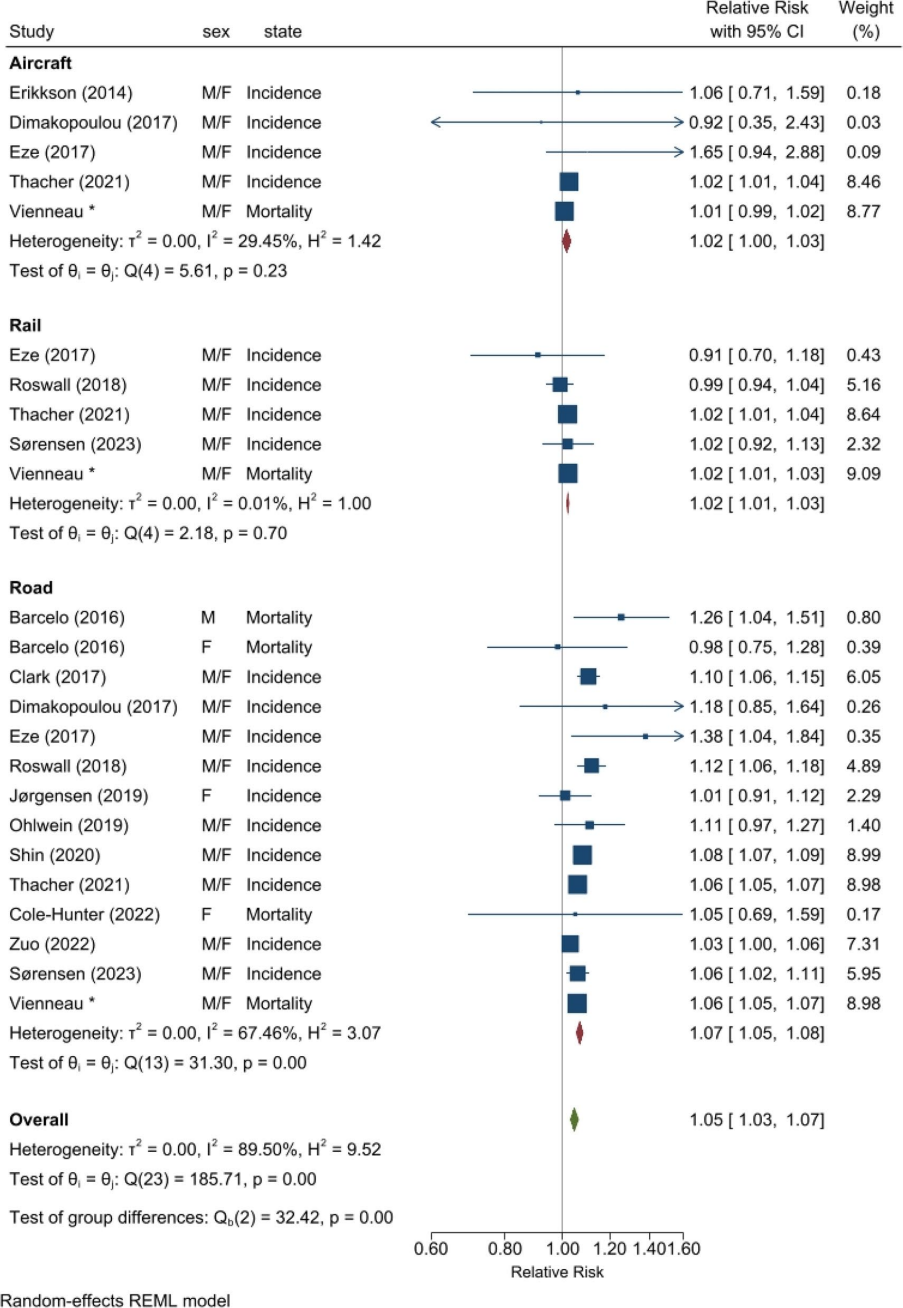
Meta-analysis for association between source-specific transportation noise and diabetes incidence and mortality combined. Allowing multiple different cohorts per country **Notes:** Cohorts are not duplicated in this meta-analysis, but smaller detailed cohorts can overlap (in persons) with the larger administrative cohorts. This applies to studies in Denmark and Switzerland. “Vienneau*” refers to the results of this study (Table 2, Model 3 for nonT1-DM)

Meta-analysis was only possible for road traffic noise in relation to mortality (1.08 [0.99, 1.18] per 10 dB L_den_, *n* = 4) (Fig. 2). Neither of the two previous studies on mortality investigated railway or aircraft noise. Considering all 14 eligible studies (i.e., ignoring potential overlaps in population due to multiple different cohorts per country), the pooled associations were increased for all noise sources (Fig. 3). Based on 5 studies, the association for aircraft noise was borderline significant (1.02 [1.00, 1.03] per 10 dB L_den_) with low heterogeneity (I^2^ 29%). Five studies were also available for railway noise, revealing a significant association with no heterogeneity (1.02 [1.01, 1.03] per 10 dB L_den_; I^2^ 0%). The majority of studies (*n* = 14 from 13 studies) were on road traffic noise, for which the pooled association was 1.07 (1.05, 1.08) per 10 dB L_den_ and somewhat heterogeneous (I^2^ 67%). The overall association, considering all sources, was 1.05 (1.03, 1.07) per 10 dB L_den_ transportation noise, with increased heterogeneity (I^2^ 90%). Both Denmark and Switzerland had more than one cohort per strata. Retaining only the largest population study per country where multiple cohorts were available produced similar pooled associations (see Supp. Meta-analysis Sect. 1.6a Forest plot).

**Fig. 4:**
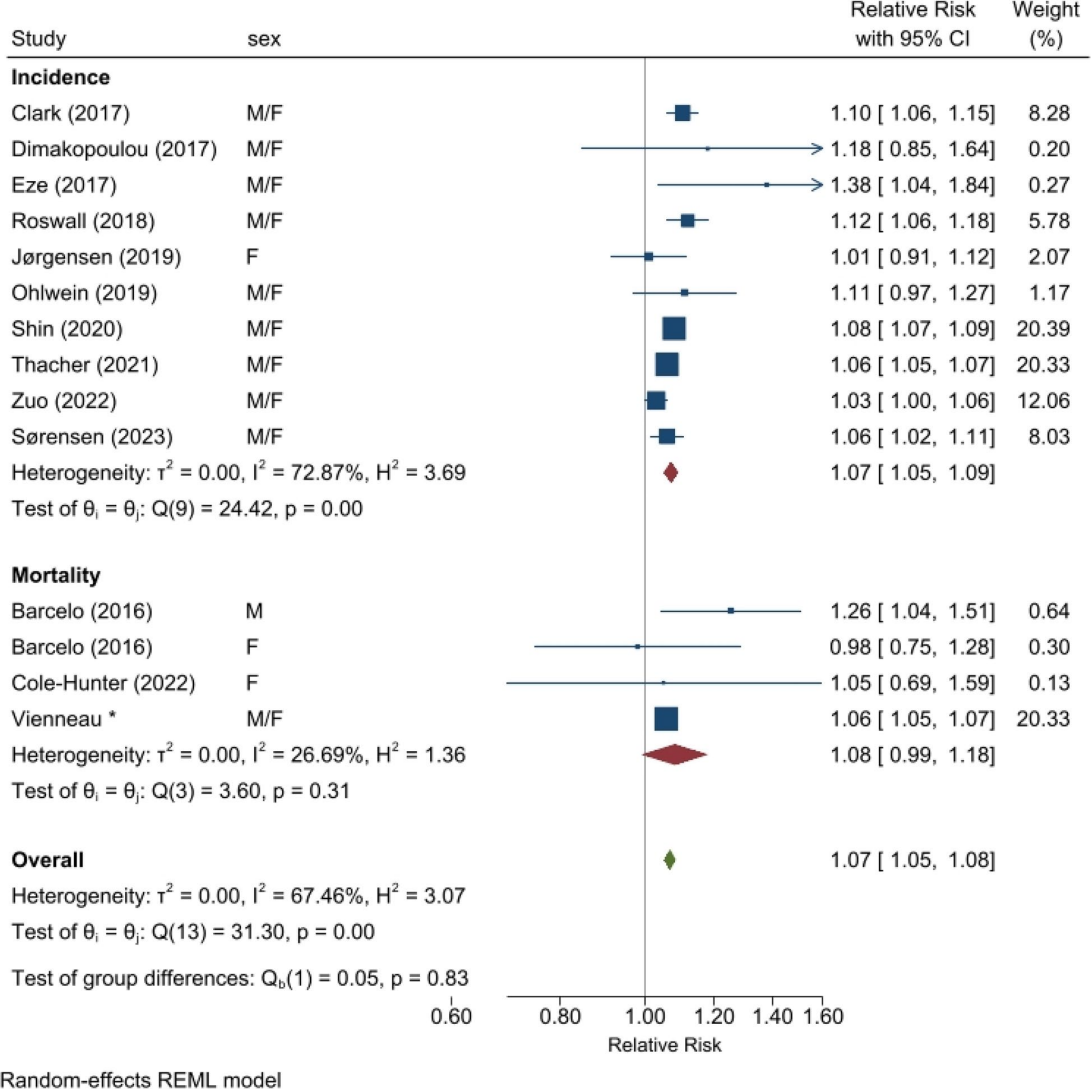
Meta-analysis for association between road traffic noise and diabetes, stratified by incidence or mortality. Allowing multiple different cohorts per country **Notes:** Cohorts are not duplicated in this meta-analysis, but smaller detailed cohorts can overlap (in persons) with the larger administrative cohorts. This applies to studies in Denmark and Switzerland.“Vienneau*” refers to the results of this study (Table 2, Model 3 for nonT1-DM)

Partitioned by disease state, the associations for road traffic noise and DM were similar for mortality (1.08 [0.99, 1.18] per 10 dB L_den_, *n* = 4 from 3 studies) and incidence (1.07 [1.05, 1.09] per 10 dB L_den_, *n* = 10), though with a slightly higher point estimate and with larger 95% confidence intervals for the former (Fig. 4). The incidence group included a larger number of studies and more heterogeneity than mortality (10 vs. 4 estimates; I^2^ 73% vs. 27%), with the mortality association dominated by the current large cohort analysis within the SNC. All point estimates were above 1.0 for the incidence studies, showing some consistency across the results despite high heterogeneity. In the sensitivity analysis, 3 Danish cohorts that overlapped with the largest by Thacher et al. [30] were dropped from the group of incidence studies; this only slightly affected the pooled estimate confidence interval (1.07 [1.05, 1.10] per 10 dB L_den_ road traffic noise) (Supp. Meta-analysis Sect. 1.6b Forest plot).

## Discussion

### Main findings

This large, national cohort study provides new evidence that diabetes mortality, in addition to incidence, is associated with exposure to transportation noise. Considering multiple noise sources and air pollution, both road traffic and railway noise were independently associated with DM mortality. Including the number of noise events at night slightly attenuated these associations. The hazard ratios for road traffic and railway noise were higher in males than females and in younger compared to older adults.

A clear association for aircraft noise was not found, except for an indication of increased risk in the mid-exposure range (between 40 and 50 dB L_den_) when restricting to the individuals that were most likely to be chronically exposed. This restriction, however, was not sufficient to fully address exposure misclassification. Specifically, the lower exposure range still included a large, heterogeneous population in terms of SES and degree of urbanisation, while those living in the higher range benefit from noise protection policies to improve windows and provide sound insulation. The nighttime flight ban during the core night hours may partly explain the lack of association, in addition to the fact that areas around airports in Switzerland tend to be higher SES potentially contributing to negative confounding.

### Comparison to previous literature

Our meta-analysis, including published incidence and mortality studies and these latest findings from Switzerland, showed the strongest association for road traffic noise (RR = 1.07 per 10 dB L_den_, with and without potential overlaps from multiple cohorts in the same country), followed by railway and aircraft noise (RR = 1.02), with the latter being only borderline significant. For road traffic noise, with the largest number of studies, the point estimate of the association with mortality was similar to that for incidence; confidence intervals however were larger due to the few studies. As per our criteria, the NOx-adjusted models were prioritized to obtain the most conservative control for traffic-related pollution.

Only two other studies specifically focused on DM mortality, with both being smaller in size than the SNC and only investigating road traffic noise. The case control study in Barcelona, including 2670 deaths (matched 1:1 with controls) and using a Bayesian approach, showed an association in men only (1.023 [1.010, 1.048] per 5 dB Lnight). Compared to the daytime noise (Lday), associations were more precise for the evening and nighttime noise exposure periods when individuals are more likely to be home [56]. The DK Nurses cohort, with 24,994 female nurses, did not find an association (1.05 [0.69, 1.58] per 10 dB 5-year mean L_den_ with a 1-year lag; a 23-year mean exposure was also null) [57]. While the DK Nurses cohort adjusted for lifestyle factors, our Swiss study could not, leaving it at risk of residual confounding. This may partly explain why we found an effect while Cole-Hunter et al. [57] did not, however, the unadjusted model in the DK Nurses cohort also reported a null association. Further, in our study statistically significant effect modification was detected for sex, with weaker associations in women than men (Table 3).

The majority of studies were on DM incidence, mainly on road traffic noise and conducted in populations within Europe. Similar to the first study on DM incidence in the DCH cohort [25] in the WHO review, Roswall et al. [28] found an association for road traffic noise of 1.12 (1.06, 1.18) per 10 dB 5-year mean L_den_ after adjusting for a range of lifestyle factors and NOx. Railway noise, however, showed no association. Incidence was also studied within the DK Nurses cohort, where an initial weak positive association was attenuated to null after adjusting for lifestyle and NO_2_ (1.01 [0.91, 1.11] per 10 dB 5-year mean L_den_) [59]. More recently, noise exposure has been linked to the DNHS, including a range of variables on lifestyle and comorbidities to study type 2 DM [61]. Specific to only a few studies, this cohort of 286,151 individuals were assigned exposure at both the minimum and maximum exposed façade. The former is generally agreed to be more relevant to noise during sleep as people would typically sleep on the quiet side should their dwelling have one. For road traffic noise, the association using the minimum exposed façade was 1.06 (1.02, 1.11) per 10 dB 10-year mean L_den_ and adjusted for PM_2.5_. The association for railway noise was null [61]. The even larger registry based DNC, similar to the SNC, that lacks lifestyle information, reported associations of 1.06 (1.05, 1.07) for road traffic noise and 1.02 (1.00, 1.03) for railway noise per 10 dB 10-year mean L_den_ at the minimum exposed facade, and adjusted for NO_2_ [30]. Aircraft noise was available in 5 dB contours and only categorical models were estimated. Thus, for integration into the meta-analysis we followed the approach by Vienneau et al. [63] to calculate a linear relationship (i.e. 1.02 [1.01, 1.04] per 10 dB 10-year mean L_den_, and adjusted for NO_2_). This is similar in magnitude as the association for aircraft noise and DM mortality found within the SNC. A multi-exposure study from Denmark was not included in the meta-analysis because it was a direct subset of the DNC. All exposures in the mutually adjusted model with noise (both minimum and maximum exposed façades), UFP, NO_2_ and green space were found to be associated with incidence of type 2 DM [64].

Otherwise the studies from Europe included the detailed Swiss Cohort Study on Air Pollution and Lung and Heart Diseases in Adults (SAPALDIA), using the same noise exposure data and multi-exposure modelling approach as in the current SNC study, that found a strong association of DM incidence with road traffic noise (1.38 [1.03, 1.83] per 10 dB L_den_) and null associations with railway and aircraft noise [27]. Likewise, the UK Biobank [62], Heinz Nixdorf Recall (HNR) study in the Ruhr Area Germany [60], and the Athens population in the Hypertension and Exposure to Noise near Airports (HYENA) study [58] showed positive associations of DM incidence for road traffic noise. No association, however, was found for aircraft noise in HYENA and the Stockholm Diabetes Prevention Program (SDPP) study [13, 58]. The two non-European studies were from Canada, specifically in Ontario [29] and in metropolitan Vancouver [26]. Both were relatively large populations showing clear associations; though as per other studies using record linkage, they could only adjust for area-level SES and lacked detailed lifestyle information. Shin et al. [29] addressed this by including comorbidities and indirect adjustment for body mass index and smoking, showing the associations to be quite consistent.

Overall the patterns of risk related to transportation noise – with the strongest association per 10 dB L_den_ for road traffic, followed by railway – and the shapes of the exposure response are similar to those for ischemic heart disease mortality previously reported in this same cohort [46]. Given the shared underlying pathophysiological mechanisms, this boosts confidence in these latest findings for DM mortality. Interestingly, this is also the first study on railway noise exposure demonstrating a link with DM mortality, albeit the association is small. As mentioned above, the few other studies that looked into railway noise and DM incidence mostly showed no associations [27, 28, 61] with the exception of Thacher et al. [30]. Noise-induced sleep disturbance is a key risk factor for DM [65], and Smith et al. [10] showed that aircraft followed by railway noise leads to the greatest sleep disturbance. An experimental study also found that eventful transportation noise at night, in particular, can decrease glucose tolerance and insulin sensitivity [14]. By nature, aircraft and railway movements lead to more intermittent noise which, if occurring at night, would imply we should see stronger effects for these sources. However, the generally weaker findings for associations between railway and aircraft noise across studies in our meta-analysis may be due to masking of low exposures by road traffic noise, or the widespread use of soundproof windows in vulnerable areas. A recent study by Olbrich et al. [66], for example, showed no association between aircraft noise and recurrent cardiovascular events in CVD patients with soundproof windows.

### Relevance and biological plausibility

DM is a serious disease with frequent complications that can impact quality of life [33]. In Switzerland, based on health care claims for the year 2011, the estimated incidence and all-cause mortality rate in patients with DM were 0.58% and 2.6%, respectively [67]. The disease is responsible for substantial health care expenditure in Europe [68], and is a major cause of mortality – worldwide, in 2021 it was estimated to be responsible for about 12% of deaths in adults [4]. In this study, DM accounts for 9.4% of all deaths during follow-up which is not insubstantial (35.9% of deaths are from all CVD [46]). This is likely an underestimation due to underreporting of DM as concomitant disease. The majority of DM-related deaths are due to secondary diseases caused by DM, such as CVD and kidney disease [69].

We suggest two possible meanings for the association of DM mortality with exposure to transportation noise. First, it could be a reflection of transportation noise increasing the risk for development of type 2 DM. This is plausible given the biological mechanisms [15, 70], negative influence of noise on physical activity [18, 19], and the robust evidence from epidemiological studies linking chronic noise exposure and incidence of type 2 DM (Fig. 2). Thus, our results may be indicative of this known association between noise exposure and DM incidence, which consequently leads to increased risk for DM mortality. The second possibility is that our findings could indicate that noise exposure has an effect on the course of disease in DM patients. Noise is known to affect glucose tolerance and dysregulate cortisol metabolism [14, 71]. Noise exposure in diabetic patients could contribute to more difficult glucose control and hence possibly worse diabetes management, increasing the risk for adverse events and eventual mortality. We found similar associations when including deaths with chronic insulin-dependent diabetes often described as juvenile diabetes. We also found an independent association in those dying with type 1 DM which supports that road traffic noise exposure influences the course of disease. We are not able to determine the exact pathway from our data, however the fact that the mortality estimates are slightly higher than incidence estimates may suggest both could be relevant.

### Strengths and limitations

Strengths of this study include the large population with little selection bias, due to the national coverage, for which detailed and source-specific noise exposure could be linked at multiple time points. On the other hand, the administrative SNC does not provide information on health-related behaviours that are likely important confounders of the relationship between noise and DM. In particular, data on BMI, diet, physical activity and sleep are not available. Further, it was not possible to completely rule out exposure misclassification for those who moved between the 2000 and 2010, since the place of residence in 2006 had to be estimated from the geocodes at the two time points and a single census variable enquiring about community of residence. Additionally, approximately 8% of individuals were dropped from the analysis to avoid potential outcome and/or exposure misclassification that would otherwise occur from a false linkage [46].

Regarding the outcome, the main limitation was that DM was exclusively defined on the basis of death certificates. A reproducibility study by Zellweger et al. [72] found the agreement between the underlying cause of death in the Swiss mortality record vs. diagnosis in the terminal hospitalization record was low for DM (kappa 0.08 to 0.04). Only 13% (or 46%) of individuals with DM as the definitive cause of death could be traced in the terminal hospital record as having DM as the principle diagnosis (or vice versa). Considering DM in any cause of death and any discharge diagnoses increased the proportion that could be traced (78% or 73%). For this reason, we considered all indications of DM as coded within the mortality record (i.e. primary as well as concomitant, consecutive or initial disease). Other studies have reported cardiovascular disease and cancer as the main cause of death in diabetes patients [73, 74]. This is also reflected in our data, for the outcome nonT1-DM, where the top three primary causes of death were from CVD (37.0%), endocrine, nutritional and metabolic disease (22.7%), and cancer (20.1%). Still we acknowledge that diabetes diagnoses for some individuals who died would have been missing, contributing to outcome misclassification. Medical and/or medication history are not included in the SNC, nor were linkages to such databases possible for the purpose of this study. The resulting non-differential outcome misclassification may produce some bias to the null but would not introduce a spurious exposure response association. Another limitation was that with only ICD codes for cause of death, specific coding for type 2 DM was not available. Thus the main analysis excluded anyone with cause of death indicated as type 1 DM.

## Conclusion

Road traffic and railway noise were associated with diabetes mortality in this large cohort in Switzerland with state-of-the-art exposure modelling, including residential history and using a multi-exposure approach. The association for aircraft noise was null, though with indications of a positive association in the mid-exposure range for the subset of individuals exposed to at least some aircraft noise at their residential location. Integrating these results into meta-analyses with published studies showed consistent, positive associations for each noise source, with the strongest associations for road traffic noise. Given the high disease prevalence, coinciding with large proportions of the population exposed to transportation noise, and recent epidemiological evidence including this study on mortality, diabetes mellitus is clearly an important outcome to be considered in the noise and health discussion.

### Supplementary Information

**Supplementary Material 1.**

## Data Availability

The SNC data cannot be shared by the authors. The data are the responsibility of the Federal Statistical Office, and may be ordered here: https://www.bfs.admin.ch/bfs/en/home/statistics/population/surveys/snc.html.
