# Supplementary material for: Long-term exposure to transportation noise and diabetes mellitus mortality: a national cohort study and updated meta-analysis
Source: Environ Health. 2024 May 4;23:46. doi: 10.1186/s12940-024-01084-0 (PMC11068573; doi:10.1186/s12940-024-01084-0)
Supplement: Supplementary file 1 — Supplementary Material 1. [file 12940_2024_1084_MOESM1_ESM.docx]

**Supplement:**

**Long-term exposure to transportation noise and diabetes mellitus mortality: a national cohort study and updated meta-analysis**

Danielle Vienneau^1,2^, Benedikt Wicki^1,2^, Benjamin Flückiger^1,2^, Beat Schäffer^3^, Jean Marc Wunderli^3^, Martin Röösli^1,2^ for the SNC study group

^1^ Swiss Tropical and Public Health Institute, Allschwil, Switzerland

^2^ University of Basel, Basel, Switzerland

^3^ Empa, Swiss Federal Laboratories for Materials Science and Technology, Laboratory for Acoustics/Noise Control, Dübendorf, Switzerland.

Contents

[Table S1. Selection of study population 2](#_Toc163830286)

[Table S2. Association between noise eventfulness at night with diabetes mortality 3](#_Toc163830287)

[Table S3. Comparison of linear models vs. splines for nonT1-DM mortality, main model 3 4](#_Toc163830288)

[Table S4. Subset population characteristics at baseline, 2001 (those living around airports) 5](#_Toc163830289)

[Figure S1. Pearson correlation between exposure variables at 2001, 2006 and 2011 corresponding to the start of each exposure assessment period 6](#_Toc163830290)

[Figure S2. Subset analysis: Natural splines (3 df) for the association between aircraft noise and nonT1-DM mortality 7](#_Toc163830291)

[Meta-analysis 8](#_Toc163830292)

[1.1 Search string 8](#_Toc163830293)

[1.2 Inclusion/Exclusion criteria 9](#_Toc163830294)

[1.3 PRISMA diagram 10](#_Toc163830295)

[1.4 Key characteristics of included studies 11](#_Toc163830296)

[1.5 Excluded studies 13](#_Toc163830297)

[1.6 Sensitivity analysis: Meta-analyses retaining only the largest cohort per country within strata 14](#_Toc163830298)

[1.7 References 16](#_Toc163830299)

## Table S1. Selection of study population

| **Description** | **N** | **% total** | **% dropped** | **Notes** |
| --- | --- | --- | --- | --- |
| Full SNC | 12,194,229 |  |  |  |
| Baseline (in cohort 04.12.2000) | 72,80,246 |  | 0 | Full population (original sample) |
| Keep matched records between the original and extended SNC | 6,680,907 |  | 8.2 | Inconsistent data: Mismatch in probabilistic linkage 2000-2010 (lost) |
| Keep adults 30+ | 4,448,073 | 100 | 33.4 | Starting point (thus 41.6% dropped from full population baseline) |
| Shift the baseline to 01.01.2001, thus exclude those who die or emigrate in the 4 weeks before this date | 4,442,308 | 99.9 | 0.1 |  |
| Exclude invalid xy coordinates & household ID | 4,345,200 | 97.7 | 2.2 |  |
| Exclude housing type = hotels, hospitals, old persons homes | 4,233,889 | 95.2 | 2.6 |  |
| Exclude imputed deaths in original SNC | 4,233,889 | 95.2 | 0.0 |  |
| Exclude education = child or unknown | 4,146,829 | 93.2 | 2.1 |  |
| Exclude missing SSEP index | 4,144,287 | 93.2 | 0.1 |  |
| Exclude missing noise or exposure data | 4,136,220 | 93.0 | 0.2 | Population included in analysis |

## Table S2. Association between noise eventfulness at night with diabetes mortality

Full cohort including 4,136,220 adults over 30 years, followed from 2001 to 2015. Multipollutant models, adjusting for road traffic, railway and aircraft noise exposure plus NO_2_. Hazard Ratios (HR) and 95% confidence intervals (CI) by quartile.

| **Outcome^a^** | **N deaths** | **Intermittency Ratio (IR) at night**  **(Model 4.1)^b^** | | |  | **Number noise events at night**  **(Model 4.2)^b^** | | |  |
| --- | --- | --- | --- | --- | --- | --- | --- | --- | --- |
|  |  | **Q2** | **Q3** | **Q4** | **VIF^c^**  **IR; max** | **Q2** | **Q3** | **Q4** | **VIF^c^**  **#Events; max** |
| Main:  nonT1-DM | 72342 | 1.020  (1.019, 1.022) | 1.018  (1.016, 1.019) | 1.019  (1.018, 1.021) | 1.79, 3.37 | 1.002  (1.000, 1.003) | 1.018  (1.017, 1.020) | 1.051  (1.050, 1.052) | 2.66, 3.32 |
| Secondary1:  DM | 73388 | 1.022  (1.020, 1.023) | 1.017  (1.016, 1.018) | 1.020  (1.018, 1.021) | 1.79, 3.38 | 1.003  (1.002, 1.004) | 1.021  (1.020, 1.023) | 1.052  (1.050, 1.053) | 2.66, 3.32 |
| Secondary 2: T1-DM | 1046 | 1.101  (1.090, 1.113) | 0.982  (0.971, 0.992) | 1.049  (1.038, 1.060) | 1.74, 3.65 | 1.111  (1.099, 1.123) | 1.249  (1.236, 1.263) | 1.106  (1.094, 1.118) | 2.80, 3.57 |

Notes:

1. Outcome definition: nonT1-DM (ICD-10: E11-E14); DM (IDC-10: E10-E14, includes type 1 diabetes); T1-DM (ICD-10: E10, type 1 diabetes)
2. Models:

Model 3 included noise exposure (road traffic, railway and aircraft noise; Lden), strata sex and period (i.e. 2001-2005, 2006-2010, or 2011-2015), individual-level covariates civil status, education level, mother tongue, nationality and quartiles of local-SEP, area-level covariates community and regional SEP and unemployment rate, and NO_2_ exposure

Model 4.1 = Model 3 + intermittency ratio at night from all sources combined (in quartiles)

Model 4.2 = Model 3 + night-time noise events from all sources combined (in quartiles)

1. VIF = the Variance Inflation Factor for the eventfulness variable of interest (IR or #Events); and the maximum VIF across all variables in the models (consistently NO_2_ had the maximum VIF)

## Table S3. Comparison of linear models vs. splines for nonT1-DM mortality, main model 3

Full cohort including 4,136,220 adults over 30 years (and 72,342 nonT1-DM deaths), followed from 2001 to 2015. Multipollutant models, adjusting for the other two noise sources plus NO2.

| **Source** | **HR (95% CI) per 10 dB increase in Lden** | **Model AIC** | | **p-value** |
| --- | --- | --- | --- | --- |
|  |  | **Linear** | **Natural spline, 3df** |  |
| Road traffic | 1.06 (1.05, 1.07) | 1154315 | 1154287 | <0.001 |
| Railway | 1.02 (1.01, 1.03) | 1154315 | 1154316 | 0.486 |
| Aircraft | 1.01 (0.99, 1.02) | 1154315 | 1154316 | 0.452 |
| Aircraft (subset) | 1.01 (0.98, 1.03) | 297025 | 297029 | 0.858 |

Notes:

Outcome definition: nonT1-DM (ICD-10: E11-E14)

Model 3 included noise exposure (road traffic, railway and aircraft noise; Lden), strata sex and period (i.e. 2001-2005, 2006-2010, or 2011-2015), individual-level covariates civil status, education level, mother tongue, nationality and quartiles of local-SEP, area-level covariates community and regional SEP and unemployment rate, and NO_2_ exposure

## Table S4. Subset population characteristics at baseline, 2001 (those living around airports)

| **Characteristic** | **Subset Cohort** | **nonT1-DM Deaths ^c^** |
| --- | --- | --- |
| Number of participants | 1,295,546 | 21,974 |
| Person-years | 17,288,027 |  |
| Male (%) | 47.4 | 52.8 |
| Age (%) |  |  |
| 30-64 | 76.4 | 22.5 |
| 65-79 | 18.5 | 55.5 |
| 80+ | 5.1 | 22.0 |
| Mother tongue (%) |  |  |
| German and Rhaeto-Romansch | 69.8 | 78.5 |
| French | 15.1 | 12.8 |
| Italian | 4.6 | 4.9 |
| Other | 10.5 | 3.8 |
| Education (%) |  |  |
| Compulsory education or less | 21.4 | 37.4 |
| Upper secondary level | 52.0 | 48.7 |
| Tertiary level education | 26.6 | 14.0 |
| Marital status (%) ^a^ |  |  |
| Single | 14.9 | 8.9 |
| Married | 67.5 | 56.1 |
| Divorced | 9.7 | 9.8 |
| Widowed | 7.8 | 25.2 |
| Swiss nationality (%) ^a^ | 79.0 | 89.0 |
| Local-SEP (%), mean (SD) ^a,b^ | 67.9 (10.5) | 66.1 (10.1) |
| Area-SEP community (%), mean (SD) ^a^ | 67.3 (5.6) | 65.5 (6.3) |
| Area-SEP community-region (%), mean (SD) ^a^ | 0.7 (4.9) | 0.2 (5.2) |
| Area unemployment community (%), mean (SD) ^a^ | 4.0 (1.4) | 3.7 (1.3) |
| Area unemployment community-region(%), mean (SD) ^a^ | 0.0 (1.1) | 0.2 (1.0) |
| Road traffic noise, Lden (dB), mean (SD) ^a^ | 55.5 (7.8) | 56.5 (7.7) |
| Railway noise Lden (dB), mean (SD) ^a^ | 37.4 (10.3) | 37.6 (10.2) |
| Aircraft noise Lden (dB), mean (SD) ^a^ | 44.0 (7.3) | 43.3 (6.9) |
| Total noise Intermittency Ratio (IR) at night (%) ^a^ | 68.2 (19.8) | 68.0 (19.8) |
| Total noise events at night (count), mean (SD) ^a^ | 200 (217.2) | 213.8 (225.1) |
| NO_2_ concentration (μg/m^3^), mean (SD) | 28.3 (6.8) | 28.9 (6.8) |
| PM_2.5_ concentration (μg/m^3^), mean (SD) | 17.3 (1.4) | 17.4 (1.4) |

Notes:

a. census/exposure data available at multiple time points, and updated at beginning of each 5-year period; baseline values shown here

b. Quartiles of local-socioeconomic position (SEP) used in models

c. Outcome definition: nonT1-DM (ICD-10: E11-E14)

## Figure S1. Pearson correlation between exposure variables at 2001, 2006 and 2011 corresponding to the start of each exposure assessment period


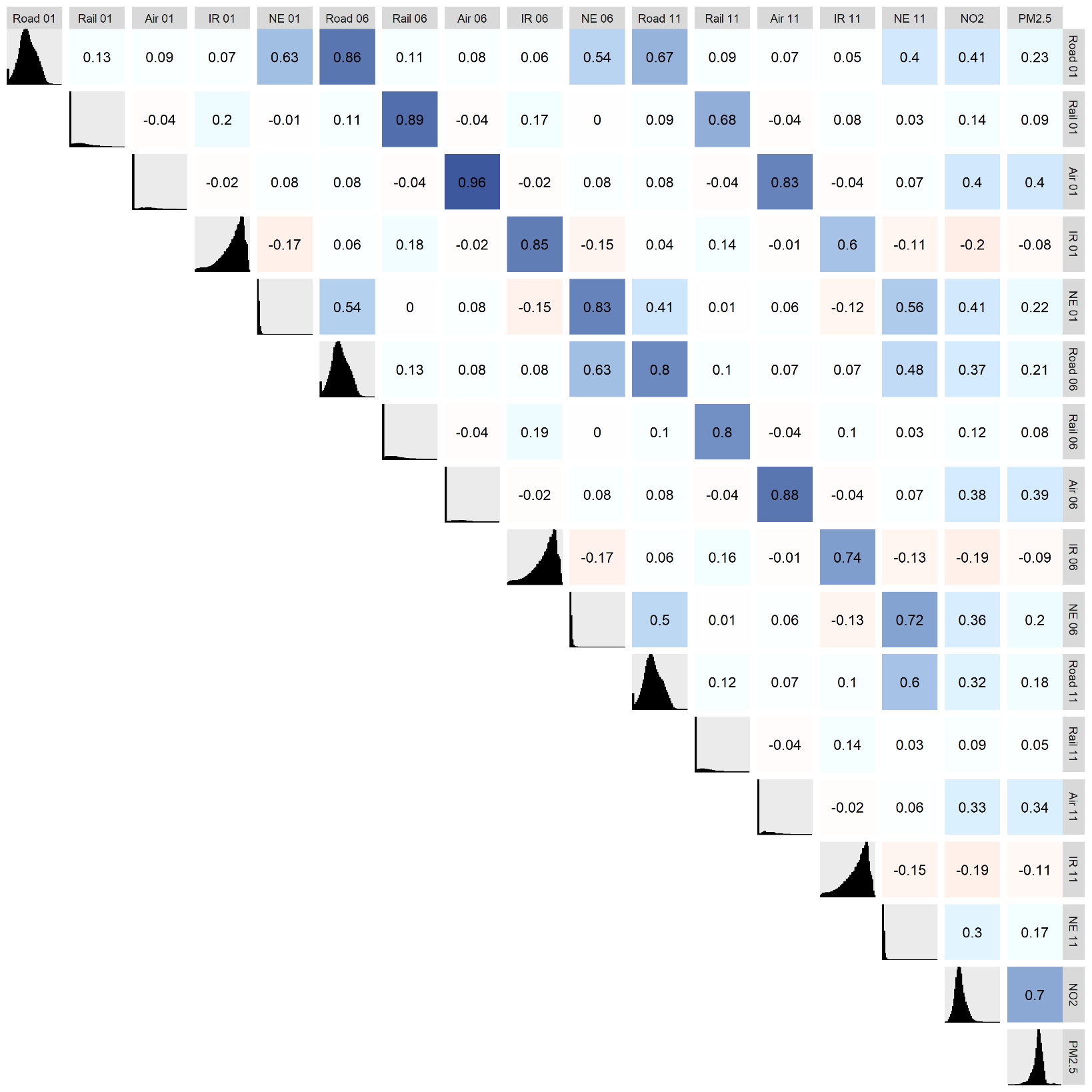


Notes: Road = road traffic noise, Rail = railway noise, Air = aircraft noise, IR = Intermittency ratio at night, NE = number of noise events at night, 01, 06 and 11 respectively refer to years 2001, 2006 and 2011.

## Figure S2. Subset analysis: Natural splines (3 df) for the association between aircraft noise and nonT1-DM mortality

Full cohort including 4,136,220 adults over 30 years (and 72,342 nonT1-DM deaths), followed from 2001 to 2015. Multipollutant models, adjusting for road traffic and railway noise plus NO2


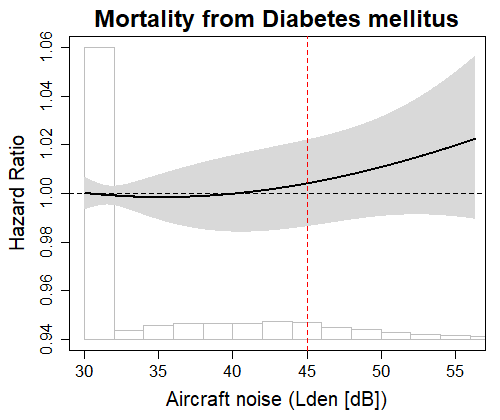


Full population

Subset around airports


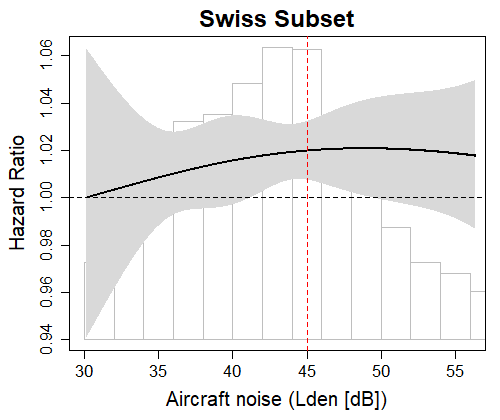


Notes:

Outcome definition: nonT1-DM (ICD-10: E11-E14)

Model 3: Natural spline (3 df) for Aircraft noise. Included strata sex and period, and adjusted for other sources of transportation noise (road traffic and railway noise), mother tongue, nationality, civil status, education, local-SEP, area-SEP and unemployment, and NO_2_

Vertical red lines show WHO guideline levels based on Lden: aircraft=45 dB

Y-axes are set to the same scale for comparison.

The “Full population” spline is the same as in Figure 1, with a rescaled y-axis.

The “Subset around airports” is the restricted analysis including only those with aircraft noise exposure > 30 dB.

## Meta-analysis

### 1.1 Search string

**Updated: 22 November 2023**

**Diabetes**

**OVID Search – adapted from van Kempen et al. (2018):**

1 ((rail* or aircraft or airport* or road* or traffic* or automobile* or vehicle*) adj5 noise.tw. *(1611)*

2 exp *Transportation/ *(51366)*

3 Aircraft/or Airports/or Railroads/or Motor Vehicles/ *(19695)*

4 *Noise/ *(13358)*

5 Noise, transportation/ *(1710)*

6 (1 or 2 or 3) and (1 or 4 or 5) *(2505)*

7 exp Diabetes Mellitus/ *(513771)*

8 exp Obesity/or exp Overweight/or exp Body Mass Index/ *(359307)*

9 (diabetes or obesit* or overweight or bmi or body mass index).tw. *(938853)*

10 7 or 8 or 9 *(1149485)*

11 6 and 10 *(128)*

12 11 not child*.ti. *(119)*

13 limit 12 to yr="2014-2019" *(54)*

14 limit 12 to yr="2014-current" *(93)*

15 limit 12 to yr="2019-current" *(49)*

### 1.2 Inclusion/Exclusion criteria

The following inclusion/exclusion criteria were applied:

a) Studies on road traffic, railway or aircraft noise exposure and incidence and mortality of diabetes (excluding gestational diabetes) were retained; those only reporting prevalence were excluded.

b) Accepted study designs were cohorts, case-control and small-area studies.

c) Exposure had to be modelled or measured. Eligible studies had to quantify the association in dB by a linear trend or in categories from which the linear trend could be calculated. RRs were expressed per 10 dB Lden prior to pooling (Vienneau et al. 2015).

d) Studies were only included if basic adjustment for socio-economic status was performed.

For eligible studies, risk estimates were extracted using the following selection criteria:

e) Modelled community noise was included only for the predominant source (e.g., if road traffic noise was the main source, risk estimates for community noise exposure were included in the road traffic noise meta-analysis).

g) If available, the risk estimates adjusted for air pollution were selected. When several models were presented in a single study, the NOx-adjusted risk estimate was selected over the PM-adjusted on the basis of NO_2_ being the better proxy for traffic related air pollution.

h) Retained studies were cross-checked against those previously included in earlier iterations of the MA (i.e. Vienneau et al. (2019) and the “start MA” by Zare Sakhvidi et al. (2018) to ensure no duplication of study populations. Where relevant, the most recent results (i.e. with larger population or longer follow-up) were retained. Replacements are noted in Table 1.3 below.

### 1.3 PRISMA diagram


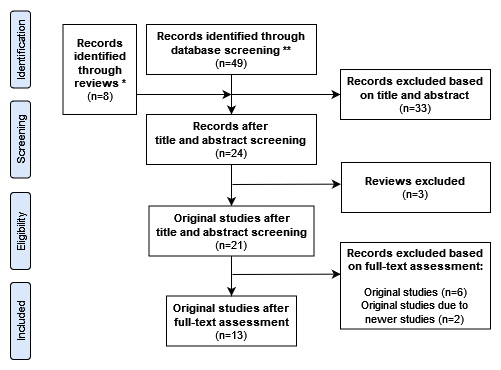


Notes:

*van Kempen et al. (2018) and Vienneau et al. (2019)

** From search conducted between 01 January 2019 and 22 November 2023, i.e. the period following the above reviews

### 1.4 Key characteristics of included studies

| **Citation**  **Cohort**  **Location ^1^** | **Status of Study in Previous reports ^2^** | **Noise source** | **Noise data** | **Original metric** | **Outcome** | **N** | **Follow-up** | **Age at baseline*** | **Sex** | **design** | **Original increment** | **Adj. other noise** | **Adj. air pollution** | **Adj. lifestyle** |
| --- | --- | --- | --- | --- | --- | --- | --- | --- | --- | --- | --- | --- | --- | --- |
| Eriksson et al. (2014)  SDPP, Sweden | Included ^a,b,c^ | Aircraft | Model | Lden | Type 2 DM  Incidence | 5156 | 1992-2006 | 47 | Both | Case  control | per 5 | - | - | - |
| Barceló et al. (2016)  Barcelona, Spain | New | Road traffic | Strategic  noise map | Lnight | DM  Mortality | 5340 (with controls) | 2004-2007 | All ages | Male  Female | Case  control | per 1 | - | NO_2_  PM_10_  Benzene | - |
| Clark et al. (2017)  BC, Canada | Included ^b,c^ | Road traffic (community) | Model | Lden | DM  Incidence | 380738 | 1999-2002 | 45-85 range | Both | Cohort | per 6.8 | - | NO | - |
| Dimakopoulou et al. (2017)  HYENA  Athens, Greece | Included ^c^ | Road traffic  Aircraft | Model | LAeq,24h (road)  Lnight (air) | DM  Incidence | 420 | 2004-2013 | 58±9.1 | Both | Cohort | per 10 | - | - | Alcohol, Smoking,  Physical activity |
| Eze et al. (2017)  SAPALDIA  Switzerland | Included ^b,c^ | Road traffic  Aircraft  Railway | Model | Lden | DM  Incidence | 2631 | 2002-2011 | 59.2± 13.1 | Both | Cohort | per 10 (road)  per 12 (air)  per 11 (rail) | Yes | NO_2_ | Alcohol, Smoking, Diet, Physical activity, Diet |
| Roswall et al. (2018)  DCH  Denmark | Previously  Sørensen et al. (2013) ^a,b^  Replaced in ^c^ | Road traffic  Railway | Model | Lden | DM  Incidence | 50534 | 1993/97-2012 | 56.2 median | Both | Cohort | per 10 | Yes | NO_x_ | Alcohol, Smoking, Diet, Physical activity, BMI |
| Ohlwein et al. (2019)  HNR  Ruhr Area, Germany | Previously  Ohlwein et al. (2017) ^b^  Replaced in ^c^ | Road traffic | Model | Lden | Type 2 DM  Incidence | 3396 | 2000-2008 | 45-74 | Both | Cohort | per 10 | - | NO_2_ | - |
| Jørgensen et al. (2019)  DK Nurses  Denmark | New | Road traffic | Model | Lden | DM  Incidence | 23762 | 1995/99-2012 | 54.0±8.3 | Female | Cohort | per 10 | - | NO_2_ | Alcohol, Smoking, Diet, Physical activity |
| Shin et al. (2020)  OPHEC  Ontario, Canada | New | Road traffic | Model | LAeq, 24h | DM  Incidence | 914607 | 2001-2015 | 55.3±14.4 | Both | Cohort | per 10 | - | NO_2_  UFP | - |
| Thacher et al. (2021)  DNC  Demnark | New | Road traffic  Aircraft  Railway | Model | Lden | Type 2 DM  Incidence | 3563991 | 2000-2017 | 52.7±14.8 | Both | Cohort | per 10 (road)  per 10 (rail)  category (air) | Yes | NO_2_ | - |
| Cole-Hunter et al. (2022)  DK Nurses  Denmark | New | Road traffic | Model | Lden | DM  Mortality | 24994 | 1995/99-2014 | 53.3±8.2 | Female | Cohort | per 10 | - | NO_2_ | Alcohol, Smoking |
| Zuo et al. (2022)  UK Biobank  United Kingdom | New | Road traffic | Model | Lden | Type 2 DM  Incidence | 305969 | 2006-2010 | 57.1±8.1 | Both | Cohort | per 10 | - | PM_2.5_ | Alcohol, Smoking Diet, BMI, sleep |
| Sorensen et al. (2023)  DNHS  Denmark | New | Road traffic  Railway | Model | Lden | Type 2 DM  Incidence | 286151 | 2010-2017 | 48.9 to 55.2  depending on source | Both | Cohort | per 10 | Yes | PM_2.5_ | Alcohol, Smoking, Physical activity, Diet |
| Vienneau*  SNC  Switzerland | New (this study) | Road traffic  Aircraft  Railway | Model | Lden | DM  Mortality | 4136220 | 2000-2015 | 30+ | Both | Cohort | per 10 | Yes | NO_2_ | - |

1. Cohort Name: BC = British Colombia; DCH = Diet, Cancer and Health Cohort; DK Nurses = Danish Nurse Cohort; DNC = Danish National Cohort; DNHS = Danish National Health Survey; OPHEC = Ontario Population Health and Environment Cohort; HNR = Heinz Nixdorf Recall; HYENA = Hypertension and Exposure to Noise near Airports; SAPALDIA = Swiss Cohort Study on Air Pollution and Lung and Heart Diseases in Adults; SDPP = Stockholm Diabetes Prevention Program; SNC = Swiss National Cohort; UK Biobank = United Kingdom Biobank

2. Status of study in previous reports: a. Studies originally included in the WHO guideline process and associated publication (van Kempen et al. 2018); b. Eligible studies included in the subset MA by Zare Sakhvidi et al. (2018); c. Included in Vienneau et al. (2019), available online at <https://edoc.unibas.ch/70857/>

* Mean age unless otherwise specified

### 1.5 Excluded studies

| **#** | **Excluded** | **Reason** |
| --- | --- | --- |
| 1 | Sørensen et al. (2013). "Long-term exposure to road traffic noise and incident diabetes: a cohort study." EHP 121(2): 217-222. | Replaced by Roswall 2018 with longer follow-up |
| 2 | Ohlwein et al. (2017). “Road traffic noise and incident diabetes mellitus after 5 years of follow-up–Results from the Heinz Nixdorf Recall Study.” Das Gesundheitswesen 79(08/09):V-153. | Replaced by Ohlwein 2019 |
| 3 | Klompmaker et al. (2019). "Associations of Combined Exposures to Surrounding Green, Air Pollution, and Road Traffic Noise with Cardiometabolic Diseases." EHP 127(8): 87003. | Outcome: diabetes prevalence |
| 4 | Huang et al. (2020). "The Association between Noise Exposure and Metabolic Syndrome: A Longitudinal Cohort Study in Taiwan." IJERPH 17(12). | Outcome: metabolic syndrome |
| 5 | So et al. (2020). "Long-term exposure to low levels of air pollution and mortality adjusting for road traffic noise: A Danish Nurse Cohort study." Env Int 143: 105983. | Air pollution study |
| 6 | Sorensen et al. (2022). "Air pollution, road traffic noise and lack of greenness and risk of type 2 diabetes: A multi-exposure prospective study covering Denmark." Env Int 170: 107570. | Already include Thacher 2021 with larger population |
| 7 | Sorensen et al. (2023). "Effects of Sociodemographic Characteristics, Comorbidity, and Coexposures on the Association between Air Pollution and Type 2 Diabetes: A Nationwide Cohort Study." EHP 131(2): 27008. | Air pollution study |
| 8 | Hu et al. (2023). "Mediation of metabolic syndrome in the association between long-term co-exposure to road traffic noise, air pollution and incident type 2 diabetes." Ecotoxicology and environmental safety 258: 114992. | Already include Zuo 2022 that has air pollution adjusted estimates |

### 1.6 Sensitivity analysis: Meta-analyses retaining only the largest cohort per country within strata

A) Meta-analysis for association between **source-specific** **transportation noise** and diabetes incidence and mortality combined. Retaining only the largest cohort per country within strata.


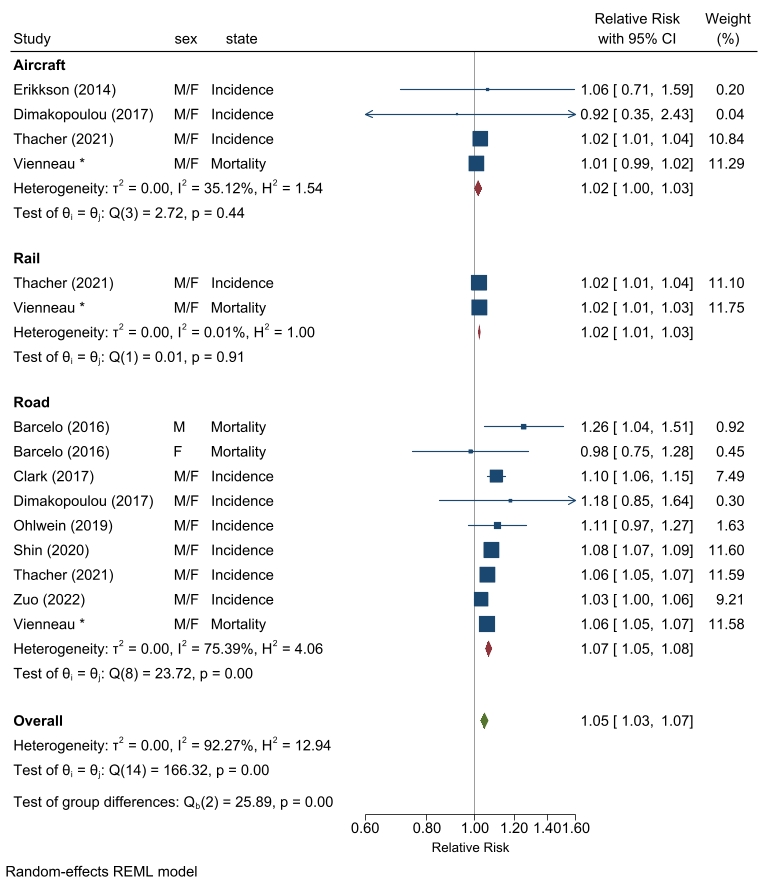


Notes:

“Vienneau*” refers to the results of this study (Table 2, Model 3 for DM –T1)

Overlapping Danish studies not included: Roswall 2018, Jørgensen 2019, Cole-Hunter 2022, and Sørensen 2023

Overlapping Swiss studies not include: Eze 2017

B) Meta-analysis for association between **road traffic noise** and diabetes, stratified by incidence or mortality. Retaining only the largest cohort per country within strata.


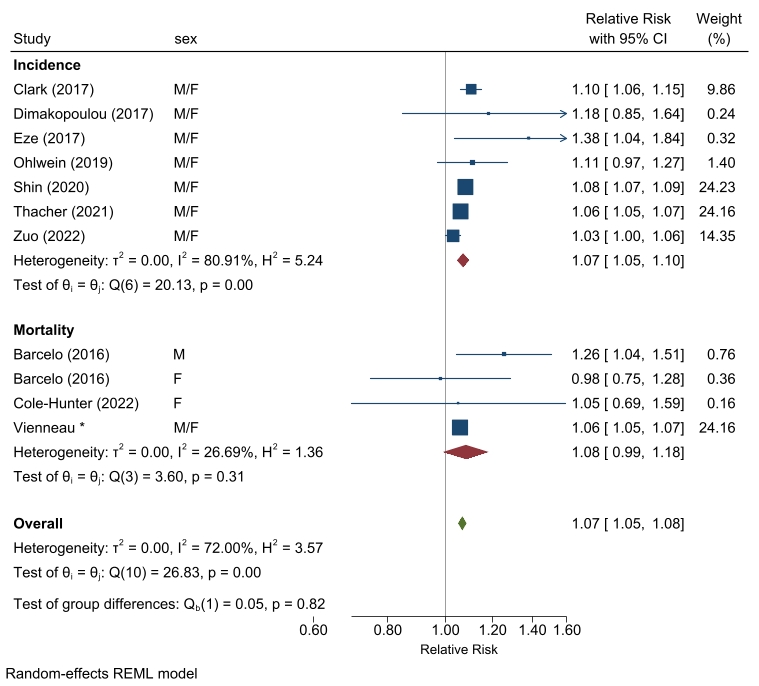


Notes:

“Vienneau*” refers to the results of this study (Table 2, Model 3 for DM –T1)

Overlapping Danish studies not included: Roswall 2018, Jørgensen 2019, and Sørensen 2023. All were incidence studies.

### 1.7 References

Barceló, M. A., D. Varga, A. Tobias, J. Diaz, C. Linares and M. Saez (2016). "Long term effects of traffic noise on mortality in the city of Barcelona, 2004-2007." Environ Res **147**: 193-206.

Clark, C., H. Sbihi, L. Tamburic, M. Brauer, L. D. Frank and H. W. Davies (2017). "Association of Long-Term Exposure to Transportation Noise and Traffic-Related Air Pollution with the Incidence of Diabetes: A Prospective Cohort Study." Environmental health perspectives **125**(8): 087025.

Cole-Hunter, T., R. So, H. Amini, C. Backalarz, J. Brandt, E. V. Bräuner, O. Hertel, S. S. Jensen, J. T. Jørgensen, M. Ketzel, J. E. Laursen, Y. H. Lim, S. Loft, A. Mehta, L. H. Mortensen, M. K. Simonsen, T. Sisgaard, R. Westendorp and Z. J. Andersen (2022). "Long-term exposure to road traffic noise and all-cause and cause-specific mortality: a Danish Nurse Cohort study." Sci Total Environ **820**: 153057.

Dimakopoulou, K., K. Koutentakis, I. Papageorgiou, M.-I. Kasdagli, A. S. Haralabidis, P. Sourtzi, E. Samoli, D. Houthuijs, W. Swart, A. L. Hansell and K. Katsouyanni (2017). "Is aircraft noise exposure associated with cardiovascular disease and hypertension? Results from a cohort study in Athens, Greece." Occupational and environmental medicine **74**(11): 830-837.

Eriksson, C., A. Hilding, A. Pyko, G. Bluhm, G. Pershagen and C. G. Ostenson (2014). "Long-term aircraft noise exposure and body mass index, waist circumference, and Type 2 diabetes: A prospective study." Environmental Health Perspectives **122**(7): 687-694.

Eze, I. C., M. Foraster, E. Schaffner, D. Vienneau, H. Héritier, F. Rudzik, L. Thiesse, R. Pieren, M. Imboden, A. von Eckardstein, C. Schindler, M. Brink, C. Cajochen, J. M. Wunderli, M. Röösli and N. Probst-Hensch (2017). "Long-term exposure to transportation noise and air pollution in relation to incident diabetes in the SAPALDIA study." Int J Epidemiol **46**(4): 1115-1125.

Hu, X., T. Yang, Z. Xu, J. Jin, J. Wang, S. Rao, G. Li, Y. S. Cai and J. Huang (2023). "Mediation of metabolic syndrome in the association between long-term co-exposure to road traffic noise, air pollution and incident type 2 diabetes." Ecotoxicology and environmental safety **258**: 114992.

Huang, T., T.-C. Chan, Y.-J. Huang and W.-C. Pan (2020). "The Association between Noise Exposure and Metabolic Syndrome: A Longitudinal Cohort Study in Taiwan." International journal of environmental research and public health **17**(12).

Jørgensen, J. T., E. V. Bräuner, C. Backalarz, J. E. Laursen, T. H. Pedersen, S. S. Jensen, M. Ketzel, O. Hertel, S. N. Lophaven, M. K. Simonsen and Z. J. Andersen (2019). "Long-Term Exposure to Road Traffic Noise and Incidence of Diabetes in the Danish Nurse Cohort." Environ Health Perspect **127**(5): 57006.

Klompmaker, J. O., N. A. H. Janssen, L. D. Bloemsma, U. Gehring, A. H. Wijga, C. van den Brink, E. Lebret, B. Brunekreef and G. Hoek (2019). "Associations of Combined Exposures to Surrounding Green, Air Pollution, and Road Traffic Noise with Cardiometabolic Diseases." Environ Health Perspect **127**(8): 87003.

Ohlwein, S., F. Hennig, S. Lucht, C. Matthiessen, N. Pundt, S. Moebus, K.-H. Jöckel and B. Hoffmann (2019). "Indoor and outdoor road traffic noise and incident diabetes mellitus: Results from a longitudinal German cohort study." Environmental Epidemiology **3**(1): e037.

Ohlwein, S., F. Hennig, S. Lucht, S. Moebus, K. Jöckel and B. Hoffmann (2017). "Road traffic noise and incident diabetes mellitus after 5 years of follow-up–Results from the Heinz Nixdorf Recall Study." Das Gesundheitswesen **79**(08/09): V-153.

Roswall, N., O. Raaschou-Nielsen, S. S. Jensen, A. Tjonneland and M. Sorensen (2018). "Long-term exposure to residential railway and road traffic noise and risk for diabetes in a Danish cohort." Environmental research **160**: 292-297.

Shin, S., L. Bai, T. H. Oiamo, R. T. Burnett, S. Weichenthal, M. Jerrett, J. C. Kwong, M. S. Goldberg, R. Copes, A. Kopp and H. Chen (2020). "Association Between Road Traffic Noise and Incidence of Diabetes Mellitus and Hypertension in Toronto, Canada: A Population-Based Cohort Study." J Am Heart Assoc **9**(6): e013021.

So, R., J. T. Jørgensen, Y. H. Lim, A. J. Mehta, H. Amini, L. H. Mortensen, R. Westendorp, M. Ketzel, O. Hertel, J. Brandt, J. H. Christensen, C. Geels, L. M. Frohn, T. Sisgaard, E. V. Bräuner, S. S. Jensen, C. Backalarz, M. K. Simonsen, S. Loft, T. Cole-Hunter and Z. J. Andersen (2020). "Long-term exposure to low levels of air pollution and mortality adjusting for road traffic noise: A Danish Nurse Cohort study." Environ Int **143**: 105983.

Sørensen, M., Z. J. Andersen, R. B. Nordsborg, T. Becker, A. Tjonneland, K. Overvad and O. Raaschou-Nielsen (2013). "Long-term exposure to road traffic noise and incident diabetes: a cohort study." Environmental Health Perspectives **121**(2): 217-222.

Sorensen, M., U. A. Hvidtfeldt, A. H. Poulsen, L. C. Thygesen, L. M. Frohn, J. Khan and O. Raaschou-Nielsen (2023). "Long-term exposure to transportation noise and risk of type 2 diabetes: A cohort study." Environmental research **217**: 114795.

Sorensen, M., A. H. Poulsen, U. A. Hvidtfeldt, J. Brandt, L. M. Frohn, M. Ketzel, J. H. Christensen, U. Im, J. Khan, T. Munzel and O. Raaschou-Nielsen (2022). "Air pollution, road traffic noise and lack of greenness and risk of type 2 diabetes: A multi-exposure prospective study covering Denmark." Environment international **170**: 107570.

Sorensen, M., A. H. Poulsen, U. A. Hvidtfeldt, J. H. Christensen, J. Brandt, L. M. Frohn, M. Ketzel, C. Andersen, V. H. Valencia, C. F. Lassen and O. Raaschou-Nielsen (2023). "Effects of Sociodemographic Characteristics, Comorbidity, and Coexposures on the Association between Air Pollution and Type 2 Diabetes: A Nationwide Cohort Study." Environmental health perspectives **131**(2): 27008.

Thacher, J. D., A. H. Poulsen, U. A. Hvidtfeldt, O. Raaschou-Nielsen, J. Brandt, C. Geels, J. Khan, T. Münzel and M. Sørensen (2021). "Long-Term Exposure to Transportation Noise and Risk for Type 2 Diabetes in a Nationwide Cohort Study from Denmark." Environ Health Perspect **129**(12): 127003.

van Kempen, E., M. Casas, G. Pershagen and M. Foraster (2018). "WHO Environmental Noise Guidelines for the European Region: A Systematic Review on Environmental Noise and Cardiovascular and Metabolic Effects: A Summary." Int J Environ Res Public Health **15**(2).

Vienneau, D., I. Eze, N. Probst-Hensch and M. Röösli (2019). Association between transportation noise and cardio-metabolic diseases: an update of the WHO meta-analysis. PROCEEDINGS of the 23rd International Congress on Acoustics. Aachen, Germany.

Vienneau, D., C. Schindler, L. Perez, N. Probst-Hensch and M. Roosli (2015). "The relationship between transportation noise exposure and ischemic heart disease: A meta-analysis." Environ Res **138**: 372-380.

Zare Sakhvidi, M. J., F. Zare Sakhvidi, A. H. Mehrparvar, M. Foraster and P. Dadvand (2018). "Association between noise exposure and diabetes: A systematic review and meta-analysis." Environ Res **166**: 647-657.

Zuo, L., X. Chen, M. Liu, L. Chen, W. Xu, H. Chen, S. Dong, Y. Wei, L. Li, S. Peng and G. Hao (2022). "Road Traffic Noise, Obesity, and the Risk of Incident Type 2 Diabetes: A Cohort Study in UK Biobank." International journal of public health **67**: 1605256.
